# Atraumatic Subcapital Femoral Neck Fracture as the Initial Presentation of Osteonecrosis of the Femoral Head: A Case‐Based Systematic Review

**DOI:** 10.1111/os.70445

**Published:** 2026-10-30

**Authors:** Komson Plangsiri, Wittawat Boonyanuwat, Pinkawas Kongmalai

**Affiliations:** ^1^ Department of Orthopedics, Faculty of Medicine Srinakharinwirot University Nakhon Nayok Thailand; ^2^ Department of Orthopedics, Faculty of Medicine Kasetsart University Bangkok Thailand

**Keywords:** avascular necrosis, femoral neck fracture, hemiarthroplasty, histopathology, osteonecrosis, PRISMA, subcapital fracture, systematic review

## Abstract

**Objective:**

Atraumatic subcapital femoral neck fracture associated with osteonecrosis of the femoral head (ONFH) is rare and may be difficult to recognize before surgery, while the available evidence regarding its diagnosis and management remains limited. We therefore aimed to describe an atypical presentation of ONFH as an atraumatic subcapital femoral neck fracture and to synthesize the available evidence on its diagnostic features, treatment patterns, and outcomes through a systematic review.

**Methods:**

A CARE‐compliant case report was combined with a PRISMA 2020–guided systematic review registered in PROSPERO. MEDLINE, Embase, Cochrane CENTRAL, and Scopus were searched from inception to February 28, 2026, for reports of atraumatic subcapital or transcervical femoral neck fractures associated with ONFH. Two reviewers independently screened studies, extracted data, and assessed methodological quality using the appropriate Joanna Briggs Institute critical appraisal checklists. Findings were synthesized descriptively because of substantial clinical heterogeneity and the absence of comparative studies.

**Results:**

Eighteen studies involving 50 patients (58 fractured hips) were included. Magnetic resonance imaging (MRI) was reported in 38 of 50 patients (76%). Reported primary‐treatment failures occurred in 3 of 8 internally fixed hips and 1 of 41 hips treated with arthroplasty; however, these uncontrolled descriptive findings do not permit comparative or causal inference.

**Conclusions:**

ONFH should be considered in atraumatic femoral neck fractures with atypical radiographic features. The available evidence is insufficient to determine the comparative effectiveness of arthroplasty and internal fixation, and treatment decisions should therefore remain individualized.

## Graphical abstract

Atraumatic subcapital femoral neck fracture can be an early presentation of osteonecrosis of the femoral head. MRI should be considered when atypical radiographic features are present, and primary arthroplasty may be associated with fewer early failures than internal fixation in suspected cases.

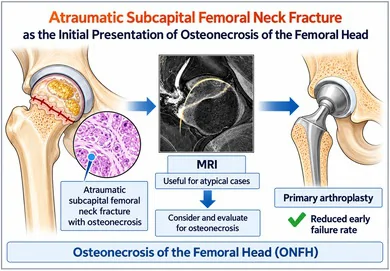

## 1 Introduction

Osteonecrosis of the femoral head (ONFH) is an ischemic bone condition caused by the disruption of blood flow to the femoral head, resulting in gradual osteocyte death and structural deterioration [1, 2]. Non‐traumatic ONFH is predominantly linked to systemic corticosteroid use, excessive alcohol intake, hemoglobinopathies, and coagulopathies; however, a significant number of cases are idiopathic [1, 3]. In the United States, an estimated 10,000–20,000 new cases of ONFH are diagnosed annually, with the disease predominantly affecting adults in their 30s to 50s [3].

The natural history of untreated ONFH follows a well‐characterized sequence: ischemic insult, bone cell death, failed repair, subchondral fracture (crescent sign), femoral head collapse, and eventually secondary osteoarthritis [2, 4]. Several classification systems have been developed to stage disease progression, of which the Association Research Circulation Osseous (ARCO) classification—revised in 2019—is among the most widely used [5]. The Ficat system, though older, remains frequently referenced in clinical practice [6].

An uncommon but clinically consequential complication occurs when extensive osteonecrosis extends into the femoral neck, weakening bone at the necrotic–viable interface and predisposing to spontaneous subcapital fracture [7, 8, 9]. These fractures may be radiographically atypical, and if the underlying osteonecrosis is not recognized, internal fixation may be attempted with a high risk of failure [7, 9, 10]. Importantly, ONFH may go entirely unrecognized prior to surgery, with the diagnosis only established on histopathological examination of the resected femoral head. To date, the evidence base for this entity consists predominantly of case reports and small case series, and no prior systematic review has been conducted using contemporary reporting guidelines.

The objectives of this manuscript are twofold: (1) to present a case of atraumatic subcapital femoral neck fracture in which ONFH was unsuspected preoperatively and confirmed only on histopathology, reported in accordance with the CARE guideline [11]; and (2) to conduct a systematic review of the published literature on atraumatic femoral neck fractures associated with ONFH, reported in accordance with the PRISMA 2020 statement [12, 13].

## 2 Case Report

### 2.1 Case Report Methods

This case is reported in accordance with the CARE guideline [11]. Written informed consent for publication, including permission to reproduce clinical images, was obtained from the patient. Formal ethical approval was not required for this case report in accordance with institutional policy.

### 2.2 Clinical Presentation

A 78‐year‐old woman with a body mass index of 33.1 kg/m^2^ presented with sudden left groin pain and an inability to bear weight. She denied any fall, direct trauma, or high‐energy mechanism. She reported a 3‐day history of progressive left hip and groin pain that began suddenly and progressed to an inability to bear weight.

Her medical history was unremarkable. She denied prior systemic corticosteroid exposure, radiation therapy, chemotherapy, sickle cell disease, heavy alcohol consumption, or known coagulopathy. She had no prior history of hip symptoms or musculoskeletal disease. Family history was unremarkable for metabolic bone disease.

On examination, the left lower limb was shortened and externally rotated. Passive hip motion elicited severe pain. Neurovascular examination of the distal limb was intact.

### 2.3 Investigations

Laboratory values, including the complete blood count, C‐reactive protein, erythrocyte sedimentation rate, serum calcium, phosphate, alkaline phosphatase, 25‐hydroxyvitamin D, blood glucose, and serum creatinine, were within the reference ranges. Serum total cholesterol was 248 mg/dL and low‐density lipoprotein cholesterol was 166 mg/dL, both above the reference range.

An anteroposterior pelvic radiograph demonstrated a displaced subcapital femoral neck fracture of the left hip, classified as Garden Type IV [14]. The fracture line was predominantly vertical, extending into the femoral head, with associated sclerosis and subchondral bone collapse, raising suspicion of an underlying pathological process (Figure 1).

**FIGURE 1:**
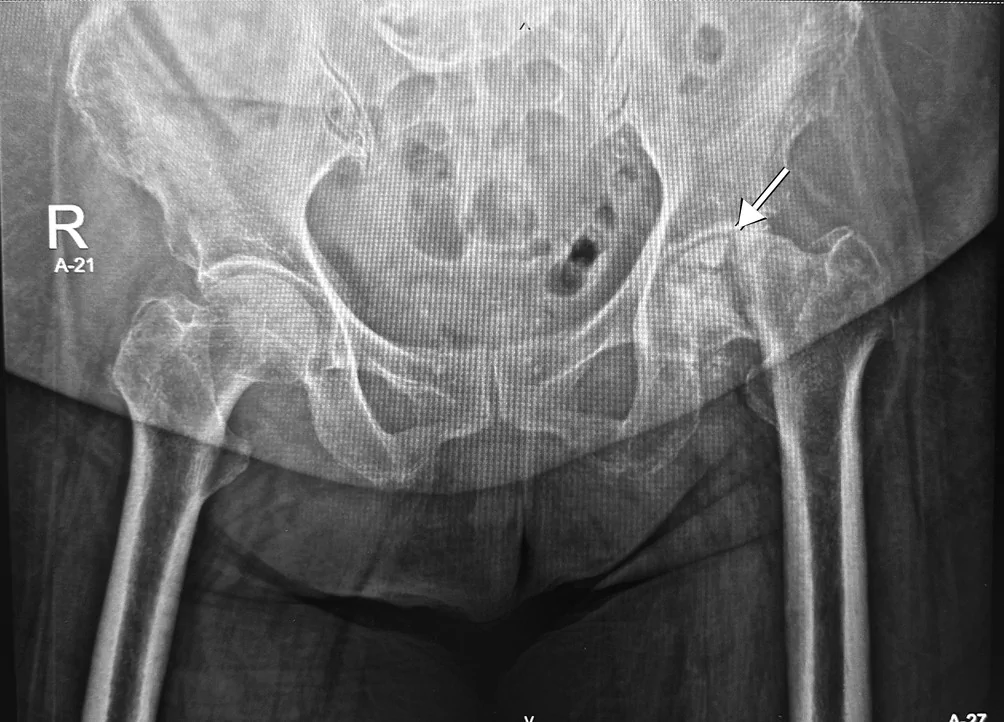
Anteroposterior pelvic radiograph obtained at presentation in a 78‐year‐old woman with atraumatic left hip pain, demonstrating a displaced subcapital femoral neck fracture (Garden Type IV). The fracture line extends vertically into the femoral head, with associated sclerotic margins and subchondral collapse, raising suspicion of an underlying pathological process.

Preoperative pelvic magnetic resonance imaging (MRI) was not performed. The atypical radiographic features (sclerotic margins, subchondral collapse, and vertical fracture line extending into the head) would ordinarily warrant further assessment with MRI before surgical intervention. Nevertheless, the clinical decision at that time prioritized immediate surgical intervention owing to the patient's age, the displaced fracture, and the severity of pain. The absence of MRI represents a significant diagnostic limitation in this context.

### 2.4 Treatment

Given the displaced subcapital fracture with extension into the femoral head in a 78‐year‐old patient, internal fixation was considered to carry a high risk of nonunion and implant failure [7, 9]. The patient underwent left hemiarthroplasty via a posterior approach using an uncemented porous‐coated femoral stem Avenir (Zimmer Biomet, Warsaw, IN, USA) with a 28‐mm cobalt‐chromium bipolar femoral head. Intraoperatively, the femoral head demonstrated grossly hemorrhagic areas with necrotic tissue, raising the intraoperative suspicion of osteonecrosis (Figure 2). The contralateral hip was asymptomatic, and clinical and radiographic surveillance was planned.

**FIGURE 2:**
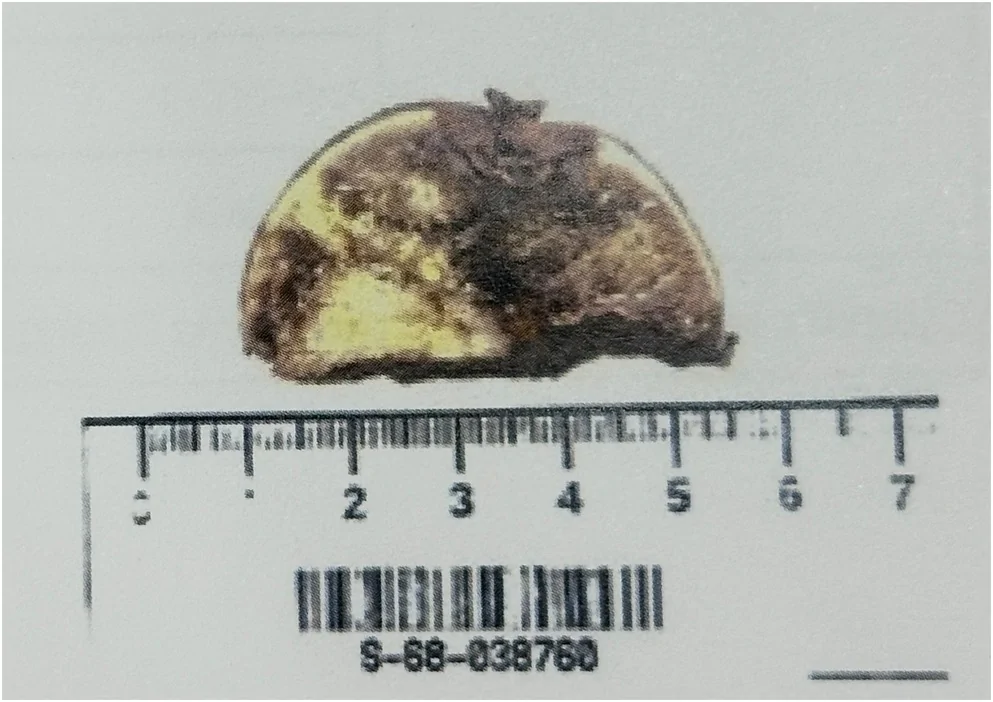
Gross photograph of the resected left femoral head obtained during hemiarthroplasty. Hemorrhagic and necrotic areas are visible, with subchondral collapse and separation of the articular cartilage from the underlying subchondral bone.

### 2.5 Histopathological Findings

The resected femoral head showed subchondral collapse and separation of articular cartilage from the subchondral bone. Significant areas of fat and bone necrosis were observed. Calcification and fibrosis were observed throughout the specimen. Bony trabeculae at the fracture site exhibited disorganization accompanied by intervening fibrosis. The articular surface exhibited localized irregularities. No evidence of malignancy, infection, or metabolic bone disease was identified. The diagnosis of extensive ONFH was confirmed as the cause of the pathological fracture (Figure 3).

**FIGURE 3:**
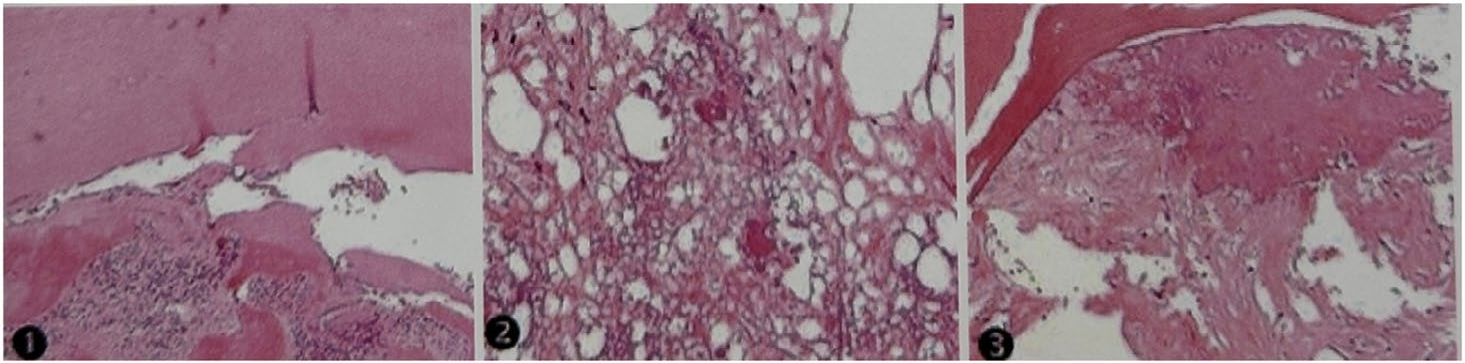
Histopathological findings of the resected femoral head with hematoxylin and eosin staining. (1) Low‐power view demonstrating separation of the articular cartilage from the subchondral bone. (2, 3) Higher‐power views of the fracture region showing extensive fat and bone necrosis, subchondral collapse, fibrosis, and trabecular disorganization. No evidence of malignancy was identified. These findings were consistent with extensive osteonecrosis of the femoral head.

### 2.6 Follow‐Up and Outcomes

The postoperative course was uncomplicated. The patient began partial weight bearing with a walker on postoperative day 2. Range of motion exercises and muscle strengthening were performed under physiotherapy guidance.

At 6‐month follow‐up, the patient reported no left hip pain, was ambulatory without walking aids, and had returned to all activities of daily living. The Harris Hip Score for the operated left hip was 83. Radiographs indicated a securely positioned and properly aligned left implant, with no signs of loosening, subsidence, or periprosthetic osteolysis (Figure 4). The contralateral right hip remained asymptomatic, with no radiographic abnormalities. Given the histopathological confirmation of ONFH and the known bilaterality of non‐traumatic ONFH (40%–80% of cases) [3], MRI of the contralateral hip was recommended as part of ongoing surveillance but was declined by the patient. The patient was surprised by the diagnosis of osteonecrosis because she had been unaware of any pre‐existing hip condition before the fracture. She was satisfied with her functional recovery and absence of pain at 6 months. The clinical course from symptom onset through the 6‐month follow‐up is summarized in Table 1.

**FIGURE 4:**
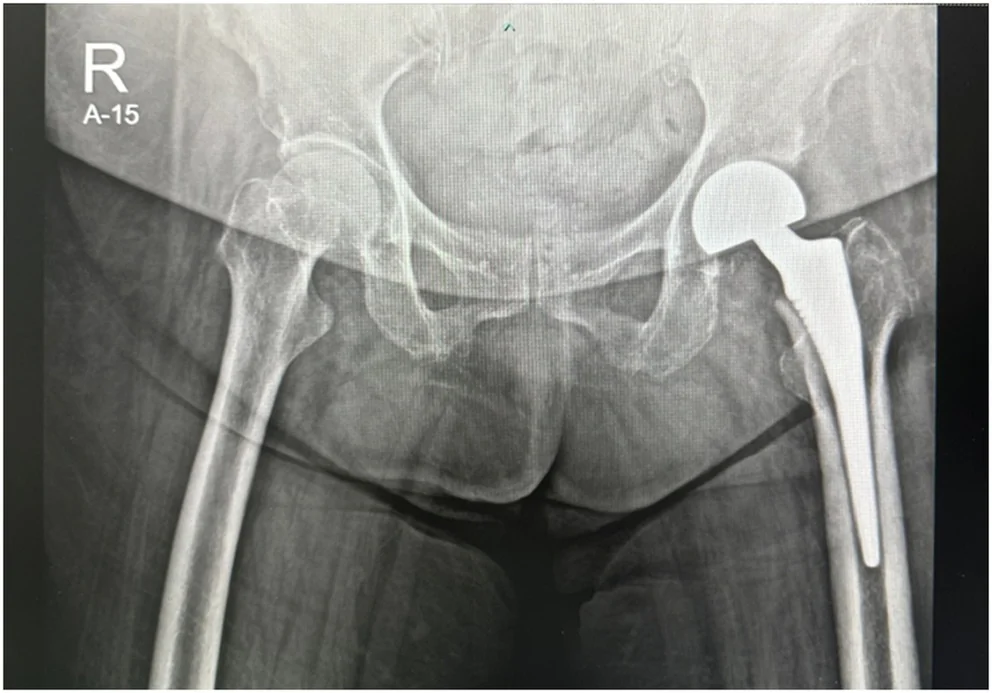
Anteroposterior pelvic radiograph obtained at six‐month follow‐up after left hemiarthroplasty, demonstrating a well‐positioned and stable femoral component without radiographic evidence of loosening, subsidence, or periprosthetic osteolysis.

**TABLE 1:** Clinical timeline of the present case.

Time point	Events
3 days pre‐presentation	Sudden onset of left groin pain while rising from a seat; progressive difficulty weight‐bearing
Day of presentation	Inability to stand; left groin tenderness; radiographs: displaced left subcapital femoral neck fracture (Garden IV) with sclerotic margins and subchondral collapse
Day 3	Admission and preoperative evaluation; MRI not obtained
Day 6	Left hemiarthroplasty; intraoperative finding of grossly necrotic femoral head
Day 8	Ambulation with walker (partial weight‐bearing)
Day 14	Suture removal; uncomplicated healing
Histopathology result	Confirmed extensive ONFH: fat and bone necrosis, subchondral collapse, fibrosis, trabecular disorganization; no malignancy
3 months	Ambulatory without gait aid
6 months	Pain‐free; Harris Hip Score, 83 (left hip); stable implant radiographically; contralateral hip MRI recommended but patient refused the MRI

## 3 Systematic Review

### 3.1 Methods

#### 3.1.1 Protocol and Registration

This systematic review was conducted in accordance with the PRISMA 2020 statement [12] and its explanation and elaboration document [13]. The protocol was registered in PROSPERO 2026 CRD420261342816.

#### 3.1.2 Eligibility Criteria

Studies were eligible if they described human subjects with radiologically and/or histologically confirmed ONFH and a concomitant or subsequent atraumatic subcapital or transcervical femoral neck fracture. Exclusion criteria included (a) femoral neck fractures that preceded and caused secondary ONFH; (b) high‐energy trauma; (c) malignancy, infection, or Paget disease; (d) conference abstracts, editorials, or reviews without new cases. No language restriction was applied.

#### 3.1.3 Information Sources and Search Strategy

We searched MEDLINE (via PubMed), Embase, Cochrane CENTRAL, and Scopus from inception to February 28, 2026. The search combined terms for osteonecrosis with terms for femoral neck fracture and atraumatic modifiers. The complete search strategy is presented in Appendix S1. Reference lists of included studies were hand‐searched.

#### 3.1.4 Study Selection

KP and WB independently screened titles and abstracts and assessed full‐text eligibility. Disagreements were resolved through discussion, and when consensus could not be reached, the final decision was made by PK. Inter‐reviewer agreement was assessed using Cohen's kappa statistic.

#### 3.1.5 Data Extraction and Risk of Bias Assessment

KP and WB independently extracted data and assessed risk of bias. Any discrepancies were resolved through discussion, with final adjudication by PK when necessary. Methodological quality was appraised using Joanna Briggs Institute (JBI) Critical Appraisal Checklists for case reports (8 items), case series (10 items), and retrospective cohort studies (11 items) [15]. Each item was rated as yes, no, unclear, or not applicable. Items rated as not applicable were excluded from the denominator when calculating the proportion of criteria met. Studies meeting more than 70% of applicable criteria were considered high quality, those meeting 50%–70% were considered moderate quality, and those meeting less than 50% were considered low quality. Inter‐rater agreement for risk of bias was substantial (Cohen's kappa = 0.82), and disagreements were resolved by consensus.

The overall certainty of evidence was assessed narratively because of the predominance of non‐comparative case reports and case series. A formal GRADE assessment was not undertaken because the evidence base did not provide comparative effect estimates.

#### 3.1.6 Synthesis Methods

A narrative synthesis was planned a priori. Descriptive statistics were used to summarize characteristics. Meta‐analysis was not performed due to the absence of comparative studies.

### 3.2 Results

#### 3.2.1 Study Selection

The database searches retrieved 410 records: PubMed (*n* = 170), Embase (*n* = 143), Cochrane CENTRAL (*n* = 25), and Scopus (*n* = 72). After removal of 127 duplicates, 283 unique records were screened, of which 231 were excluded at the title/abstract level. Fifty‐two full‐text articles were assessed. Following final eligibility adjudication, 11 studies were included from the database search. An additional 7 studies were identified through hand‐searching, yielding 18 studies (50 patients, 58 fractured hips) (Figure 5). Cohen's kappa for study selection was 0.87.

**FIGURE 5:**
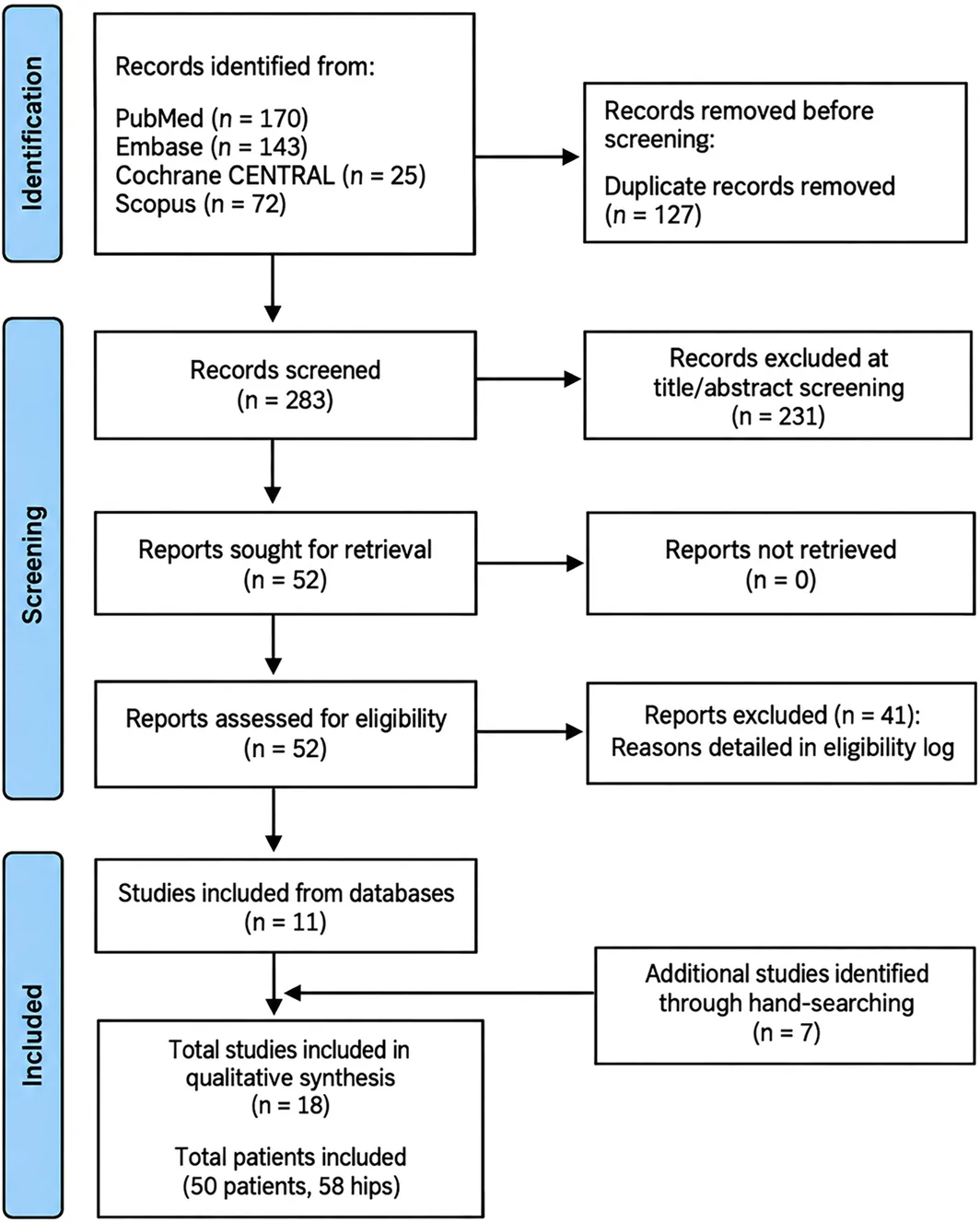
PRISMA 2020 flow diagram. Records identified: PubMed (*n* = 170), Embase (*n* = 143), Cochrane CENTRAL (*n* = 25), and Scopus (*n* = 72); total = 410. Duplicates removed (*n* = 127). Records screened (*n* = 283). Records excluded at title/abstract (*n* = 231). Full‐text assessed (*n* = 52). Studies included from databases (*n* = 11). Additional studies identified by hand‐searching (*n* = 7). Total included: 18 studies (50 patients, 58 fractured hips).

#### 3.2.2 Study Characteristics

The 18 studies were published between 1996 and 2024 in seven countries/settings. Most were case reports or small case series, with one retrospective cohort and several retrospective series contributing additional data. Study characteristics are summarized in Table 2.

**TABLE 2:** Characteristics of included studies in the final synthesis (18 studies, 50 patients, 58 fractured hips).

Study	Country/setting	Design	Patients (hips)	Sex (M/F)	MRI performed/reported	Main reported risk factor(s)	Primary treatment summary	Key outcome/analytic note
Usui et al. (1996)	Japan	Case series	4 (4)	0/4	Not reported	Steroid; SLE/ITP	Femoral head replacement/resurfacing	—
Kim and Kim (2000)	Korea	Case series	2 (2)	2/0	Yes (*n* = 2)	Alcohol 1; steroid 1	Bipolar hemiarthroplasty	—
Min et al. (2001)	Korea	Retrospective case series	10 (10)	9/1	Yes (*n* = 10)	Alcohol 8; idiopathic 2	THA	—
Loddenkemper et al. (2002)	Germany	Case report	1 (1)	0/1	Not reported	Steroid use	THA	—
Kamiya et al. (2004)	Japan	Case report	1 (2)	0/1	Yes (1 patient)	Steroid use for Sjogren syndrome	Bilateral bipolar hemiarthroplasty	—
Ko et al. (2004)	China	Case report	1 (1)	0/1	Not reported	Cushing disease/hypercortisolism	Valgus osteotomy/head preservation	Long‐term fracture union with femoral head preservation reported
Lee and Suh (2005)	Korea	Case report	1 (2)	1/0	Yes (1 patient)	Alcohol	Right THA; left in situ pinning	No primary‐treatment failure reported
Zuckerman et al. (2006)	USA	Case report	1 (2)	0/1	Yes (1 patient)	Steroid use	Bilateral SHS	Bilateral fractured hips counted separately
Tompkins et al. (2010)	Malawi	Retrospective case series	3 (3)	1/2	Not reported	HIV/ART; steroid in 1	THA x2; screw fixation x1	One failed osteosynthesis subsequently treated with THA
Vaishya (2012)	India	Case report	1 (1)	1/0	Not reported	Sickle cell disease	THA	—
Fukui et al. (2015)	Japan	Case report	1 (1)	1/0	Yes (1 patient)	Steroid use for dermatomyositis	Bipolar hemiarthroplasty	—
Kumar et al. (2016)	India	Case report	1 (1)	1/0	Yes (1 patient)	Alcohol/tobacco	THA	—
Shah and Desai (2019)	India	Case report	1 (1)	1/0	Yes (1 patient)	Alcohol	THA	Contralateral ONFH without femoral neck fracture was not counted as a fractured hip
Arora et al. (2021)	India	Retrospective case series	10 (12)	9/1	Yes (*n* = 10)	Steroid 7; alcohol 3; idiopathic 3	THA in all 12 fractured hips	Two patients had bilateral fractures
Nishino et al. (2021)	Japan	Case report	1 (2)	0/1	Yes (1 patient)	Idiopathic ONFH; low bone mineral density/vitamin D deficiency	Bilateral SHS	—
Xia et al. (2023)	China	Case report	1 (2)	1/0	Yes (1 patient)	Heavy alcohol	Bilateral THA	Bilateral fractured hips counted separately
Guo et al. (2023)	China	Retrospective cohort	8 (8)	NR	Yes (*n* = 8)	ONFH subgroup; steroid enriched	Not disaggregated for ONFH subgroup	Treatment and failure outcomes were not disaggregated; these 8 hips were excluded from treatment‐outcome pooling
Kwan et al. (2024)	China	Two‐case report	2 (3)	1/1	Not reported	Alcohol‐related risk in both cases	Cannulated screws x2; bipolar HA x1	Both screw‐fixed hips developed nonunion or implant failure; bipolar HA underwent revision for recurrent dislocation

*Note:* Patients (fractured hips) indicates the number of patients and confirmed fractured hips represented by each report. MRI counts are reported at the patient level. Bilateral ONFH without a confirmed femoral neck fracture was not counted as an additional fractured hip. Risk factors were not mutually exclusive. Guo et al. contributed to the patient, fractured‐hip, and MRI totals but were excluded from treatment‐outcome pooling because treatment and failure data were not reported separately for the ONFH subgroup.

Abbreviations: ART, antiretroviral therapy; HA, hemiarthroplasty; HIV, human immunodeficiency virus; ITP, immune thrombocytopenia; MRI, magnetic resonance imaging; NR, not reported; ONFH, osteonecrosis of the femoral head; SHS, sliding hip screw; SLE, systemic lupus erythematosus; THA, total hip arthroplasty.

#### 3.2.3 Patient Demographics and Risk Factors

Across the 50 patients, the weighted mean age was 49.3 years. Thirty‐two (64%) were male and 18 (36%) were female. Corticosteroid exposure and heavy alcohol use were the most frequently reported associations. Additional reported contexts included HIV/antiretroviral therapy, sickle cell disease, endogenous hypercortisolism/Cushing disease, low bone mineral density or osteoporosis, and idiopathic disease. Because several reports described overlapping etiologies, risk factors were synthesized narratively rather than as mutually exclusive pooled categories. Patient‐level summary data are presented in Table 3.

**TABLE 3:** Patient‐level summary of final synthesis (*N* = 50 patients).

Characteristic	Value
Age, weighted mean, years	49.3
Male sex, *n* (%)	32 (64)
Female sex, *n* (%)	18 (36)
Most frequently reported risk associations	Corticosteroid exposure; heavy alcohol consumption
Other reported associated contexts	HIV/antiretroviral therapy; sickle cell disease; endogenous hypercortisolism/Cushing disease; low bone mineral density/osteoporosis; idiopathic disease
Bilateral ONFH across reports	Frequently described, but not consistently extractable across all studies
Prodromal hip/groin pain	Commonly reported before acute fracture
MRI obtained, *n* (%)	38 (76)
Delayed ONFH recognition	Reported in several cases after surgery or after failure of initial treatment

Abbreviations: HIV, human immunodeficiency virus; MRI, magnetic resonance imaging; ONFH, osteonecrosis of the femoral head.

#### 3.2.4 Fracture and Imaging Characteristics

All 58 fractured hips occurred in the subcapital or transcervical region. MRI was reported in 38 of 50 patients (76%) and remained the most informative imaging modality when obtained. Several reports described ONFH that was initially occult or unrecognized at presentation, with the diagnosis established by further imaging or after failure of the initial treatment [8, 9, 10].

#### 3.2.5 Treatment and Outcomes

Treatment pooling was limited to 50 fractured hips for which the primary management of the ONFH‐associated fracture could be extracted. Eight ONFH‐associated hips from Guo et al. were retained in the patient and hip totals but excluded from treatment pooling because subgroup treatment was not disaggregated. Within the treatment‐extractable subset, primary arthroplasty was performed in 41 of 50 hips (82%): total hip arthroplasty in 31, hemiarthroplasty/bipolar arthroplasty in 6, and other arthroplasty procedures in 4. Internal fixation was attempted in 8 of 50 hips (16%) and one hip (2%) underwent head‐preserving osteotomy. Documented primary‐treatment failures occurred in 4 of 50 hips (8%): 1 of 41 arthroplasty hips (2%) and 3 of 8 internally fixed hips (38%). These proportions are descriptive only and should not be interpreted as comparative effectiveness estimates. Treatment outcomes are summarized in Table 4.

**TABLE 4:** Primary treatment modalities and outcomes for treatment‐extractable fractured hips (*N* = 50).

Treatment		*n* (%)	Failure, *n* (%)	Time to fracture healing/union	Follow‐up
Primary arthroplasty		41 (82)	1 (2)	Not applicable	NR/varied among reports
	THA	31 (62)	0 (0)	Not applicable	NR/varied among reports
	Hemiarthroplasty/bipolar	6 (12)	1 (17)	Not applicable	NR/varied among reports
	Other arthroplasty	4 (8)	0 (0)	—	NR/varied among reports
Primary internal fixation		8 (16)	3 (38)	Inconsistently reported	NR/varied among reports
	Cannulated screws	4 (8)	3 (75)	3 failures; exact union time for successful hip NR	Failure documented at 9 months in 2 hips; other data as reported
	Sliding hip screw	4 (8)	0 (0)	Exact union time inconsistently reported; imaging improvement at 5 months in 2 hips	≥ 5 months where reported
Head‐preserving osteotomy		1 (2)	0 (0)	Union reported; exact time NR	10 years
Total treatment‐extractable hips		50 (100)	4 (8)	—	—

*Note:* Eight ONFH‐associated fractured hips from Guo et al. were retained in the overall patient and hip totals but excluded from treatment‐outcome pooling because treatment and outcome data for the ONFH subgroup were not reported separately. Fracture‐healing time was considered not applicable for arthroplasty procedures. Where an exact time to fracture union or follow‐up duration was unavailable from the current extraction, this was designated as NR.

Abbreviations: NR, not reported; ONFH, osteonecrosis of the femoral head.

#### 3.2.6 Risk of Bias in Included Studies

Using the appropriate JBI checklists, most case reports and case series were of moderate‐to‐high methodological quality. Common deficiencies included incomplete timeline detail and limited adverse‐event reporting. The retrospective observational studies were appraised with the relevant JBI tools and provided more structured data than single‐case reports, but all included studies remained non‐comparative and at inherent risk of selection and publication bias. Details are provided in Table S1.

#### 3.2.7 Certainty of Evidence

The overall certainty of evidence was very low, reflecting the predominance of non‐comparative case reports and small case series. Formal GRADE assessment was not applied because the available evidence base lacks comparative effect estimates and therefore does not meet the standard requirements for GRADE methodology. This limitation pertains to the underlying evidence rather than to the review methods themselves. The consistent direction of findings across 18 studies from seven countries/settings over nearly three decades partially mitigates—but does not overcome—these concerns.

## 4 Discussion

### 4.1 Main Findings

This case‐based systematic review highlights atraumatic subcapital femoral neck fracture as a rare but clinically important presentation of osteonecrosis of the femoral head (ONFH). The systematic review identified 18 studies comprising 50 patients and 58 fractured hips. MRI was reported in 38 of 50 patients (76%). Among the 50 hips with extractable treatment data, arthroplasty was the most frequently reported treatment, accounting for 41 hips, whereas internal fixation was performed in eight hips and head‐preserving osteotomy in one hip. Primary treatment failure was reported in 1 of 41 hips treated with arthroplasty and 3 of 8 hips treated with internal fixation. These observations are descriptive and should not be interpreted as evidence of comparative treatment effectiveness. The present case further illustrates the diagnostic challenge of this condition, as ONFH was not recognized preoperatively and was confirmed only by histopathological examination after hemiarthroplasty.

### 4.2 Pathophysiology and Diagnostic Considerations

The proposed mechanism of femoral neck fracture in ONFH involves progressive osteoclastic resorption at the reparative interface, where incomplete remodeling may weaken the structural integrity of the femoral head–neck region [1, 2]. Histopathological findings in the present case, including extensive fat and bone necrosis, subchondral collapse, and trabecular disorganization with fibrosis at the fracture site, are consistent with this proposed mechanism.

An important diagnostic consideration is that underlying ONFH may not be apparent at the initial presentation of a femoral neck fracture. Previous reports have described ONFH that was initially occult or unrecognized, with the diagnosis established by further imaging or after failure of the initial treatment [8, 9, 10]. In the present patient, ONFH was not suspected before surgery and was identified only on histopathological examination of the resected femoral head. This finding emphasizes the importance of considering underlying ONFH when a femoral neck fracture occurs without an appropriate traumatic mechanism, particularly when atypical radiographic findings are present.

MRI has an important role in detecting and characterizing ONFH [7, 9]. Fukui et al. reported an occult femoral neck fracture associated with extensive ONFH in which conventional radiographs were initially nondiagnostic and MRI helped distinguish an ONFH‐associated pathological fracture from an insufficiency fracture [9]. In the present case, the absence of preoperative MRI prevented assessment of the extent and stage of ONFH. MRI could have demonstrated the distribution of osteonecrosis, subchondral collapse, and extension of the necrotic process toward the femoral neck, thereby increasing preoperative diagnostic certainty.

Nevertheless, preoperative MRI would not necessarily have altered the definitive surgical procedure in this patient. Given her advanced age and displaced Garden IV fracture, arthroplasty was already considered an appropriate treatment option. Thus, the principal potential benefit of MRI in this case would have been improved diagnostic certainty, assessment of disease extent, and more informed preoperative planning rather than a change in the definitive surgical treatment.

Differentiating an ONFH‐associated femoral neck fracture from an insufficiency fracture is particularly important in older patients presenting without significant trauma. Although radiographic findings such as subchondral sclerosis and femoral head collapse may provide diagnostic clues, MRI should be considered when the radiographic appearance or clinical presentation is atypical [9]. The present case demonstrates the diagnostic limitation associated with the absence of MRI but does not independently establish the clinical benefit of routine MRI in all patients with femoral neck fractures.

### 4.3 Clinical Presentation and Comparison With Previous Reports

The present patient differed from many patients described in previous reports. She was 78 years old, female, and had no identifiable conventional risk factor for ONFH. In contrast, Arora et al. reported a median age of 32 years (range, 24–61 years) among 10 patients, with corticosteroid exposure present in seven patients [7]. Corticosteroid exposure and heavy alcohol consumption were also among the most frequently reported risk factors across the studies included in the present review. These differences in age and risk‐factor profile may have contributed to the low preoperative suspicion of ONFH in the present patient.

The duration of symptoms before presentation also varied considerably in the previous literature. Arora et al. reported a mean interval of 13.8 days (range, 1–28 days) from spontaneous subcapital fracture to presentation [7]. Fukui et al. evaluated their patient 2 weeks after the onset of hip pain [9], whereas Kwan et al. described a patient with a chronic displaced femoral neck fracture following trivial trauma 2 months previously [10]. In comparison, the present patient had only 3 days of progressive hip and groin pain before becoming unable to bear weight. Thus, her three‐day presentation was shorter than the mean interval reported by Arora et al., although it remained within the range previously described [7].

The absence of conventional ONFH risk factors was another notable feature of the present case. Elevated serum cholesterol and low‐density lipoprotein cholesterol levels were observed. Abnormalities in lipid metabolism have been proposed as one of several mechanisms potentially contributing to non‐traumatic ONFH [4]; however, the significance of dyslipidemia in this individual patient remains uncertain, and no causal association can be inferred from a single case.

The major demographic, diagnostic, and treatment characteristics of the present case compared with the findings of the systematic review are summarized in Table 5.

**TABLE 5:** Case in context: Comparison of present case with final synthesis findings.

Feature	Present case	Final synthesis
Age (years)	78	49.3 (weighted mean)
Sex	Female	32/50 male (64%)
Risk factor	No conventional risk factor identified	Corticosteroid exposure and alcohol use most commonly reported
Prodromal duration	3 days	Typically described before the acute fracture event; duration variably reported
MRI obtained	No	38/50 (76%)
ONFH diagnosis timing	Postoperative histopathology	Often recognized preoperatively, but delayed diagnosis after surgery or treatment failure was also reported
Surgery	Hemiarthroplasty	Arthroplasty in 41/50 treatment‐extractable hips (82%)
Follow‐up	6 months	Variably reported across studies

Abbreviations: MRI, magnetic resonance imaging; ONFH, osteonecrosis of the femoral head.

### 4.4 Treatment Patterns and Clinical Implications

Treatment strategies varied across the included studies. Among the 50 treatment‐extractable hips, arthroplasty was performed in 41 hips, internal fixation in eight hips, and head‐preserving osteotomy in one hip. Arthroplasty therefore represented the predominant treatment strategy reported in the available literature.

Previous studies illustrate the different approaches and outcomes reported for this uncommon condition. Arora et al. treated all 12 fractured hips in their series with total hip arthroplasty and reported improvement in the mean Harris Hip Score from 10.8 preoperatively to 93 at a mean follow‐up of 25 months [7]. In contrast, Kwan et al. described bilateral femoral neck fractures treated with cannulated screw fixation that subsequently developed nonunion and implant failure at 9 months, requiring conversion to total hip arthroplasty [10].

From a biological perspective, internal fixation depends on fracture healing in bone affected by osteonecrosis, whereas arthroplasty removes the necrotic femoral head and does not depend on femoral neck union. However, the available evidence does not establish whether these biological differences account for the observed treatment outcomes. The current literature therefore does not support a conclusion regarding the superiority of one treatment strategy over another. Treatment decisions should remain individualized according to patient age, fracture morphology and displacement, bone quality, reconstructability, functional requirements, and the suspected extent of ONFH.

In the present 78‐year‐old patient, hemiarthroplasty was selected because of the displaced Garden IV fracture [14], the absence of acetabular involvement, and the goal of achieving reliable pain relief and early functional recovery. The combination of advanced age, fracture displacement, and the intraoperative finding of extensive necrotic tissue supported the decision not to pursue internal fixation. This case‐specific decision should not be interpreted as evidence that hemiarthroplasty or arthroplasty is superior to other treatment options.

### 4.5 Contralateral Hip Surveillance

Non‐traumatic ONFH is frequently bilateral, making assessment of the contralateral hip clinically relevant [3]. Bilateral ONFH and bilateral femoral neck fractures were also described in several reports included in the present review. Although the contralateral hip in the present patient remained asymptomatic and radiographically normal at 6 months, the absence of MRI does not exclude occult ONFH.

MRI of the contralateral hip was therefore recommended but was declined by the patient. Continued clinical and radiographic surveillance remains appropriate because early contralateral ONFH may remain asymptomatic.

### 4.6 Strengths, Limitations, and Future Directions

A strength of this study is the integration of a clinically informative case with a systematic synthesis of the available evidence on this uncommon presentation. The systematic review was prospectively registered in PROSPERO and conducted in accordance with the PRISMA 2020 statement [12, 13]. Study selection, data extraction, and risk‐of‐bias assessment were independently performed by two reviewers, and methodological quality was evaluated using the appropriate Joanna Briggs Institute critical appraisal checklists [15].

Several limitations should nevertheless be acknowledged. First, preoperative MRI was not obtained in the present case; therefore, the stage and extent of ONFH could not be established before surgery, and occult involvement of the contralateral hip could not be excluded. The present case illustrates the diagnostic limitations associated with the omission of MRI but does not independently establish its clinical utility.

Second, no conventional ONFH risk factor was identified in the present patient, limiting etiologic interpretation and the generalizability of this individual case.

Third, the available evidence consisted predominantly of case reports and small case series without controlled comparative studies. The included studies were clinically heterogeneous in patient age, underlying etiology, ONFH stage, fracture characteristics, treatment selection, follow‐up duration, and outcome reporting. This heterogeneity limits the generalizability of the findings and precludes reliable comparative interpretation of the observed treatment outcomes. Publication bias, selection bias, confounding by indication, and possible preferential reporting of unsuccessful internal fixation cases may also have influenced the observed failure proportions. Consequently, the treatment patterns and failure proportions reported in this review should be regarded as descriptive and hypothesis‐generating rather than as estimates of comparative effectiveness.

Fourth, ONFH stage and lesion extent were inconsistently reported and were assessed using different classification systems across the included studies. Because the extent of osteonecrosis and degree of femoral head collapse may influence both treatment selection and prognosis, the absence of standardized staging data precluded stage‐stratified analyses. Future studies should consistently use a standardized ONFH staging system, such as the revised ARCO classification [5], and report lesion extent, fracture morphology, treatment selection, time to fracture union where applicable, functional outcomes, and clearly defined follow‐up intervals.

Finally, follow‐up of the present case was limited to 6 months. This duration is insufficient to evaluate long‐term implant survival, late prosthesis‐related complications, functional durability, or the longer‐term status of the contralateral hip. Accordingly, the favorable clinical and radiographic findings observed at 6 months should be interpreted as short‐term outcomes only.

Given the rarity of this condition, multicenter observational studies with standardized diagnostic criteria, ONFH staging, fracture characterization, treatment reporting, and long‐term outcome assessment may provide more robust evidence regarding prognostic factors and treatment outcomes in the future.

## 5 Conclusions

Spontaneous subcapital femoral neck fracture is a rare but clinically important presentation of osteonecrosis of the femoral head that may remain unrecognized before surgery. ONFH should be considered when an atraumatic femoral neck fracture is accompanied by atypical radiographic features, and MRI may assist in identifying the underlying pathology and defining disease extent. Histopathological examination can provide definitive diagnostic information when the diagnosis remains uncertain.

Although different failure proportions were observed after arthroplasty and internal fixation in the available reports, the evidence is predominantly non‐comparative and does not establish the superiority of either treatment strategy. Management should therefore be individualized according to patient characteristics, fracture morphology, bone quality, reconstructability, and the extent of osteonecrosis. Because non‐traumatic ONFH may be bilateral, assessment and continued surveillance of the contralateral hip should also be considered.

## Author Contributions

**Komson Plangsiri:** conceptualization, methodology, data curation, investigation, formal analysis, writing – original draft. **Wittawat Boonyanuwat:** data curation, investigation, validation, formal analysis, resources. **Pinkawas Kongmalai:** validation, formal analysis, supervision, writing – review and editing, project administration.

## Funding

The authors have nothing to report.

## Ethics Statement

Formal ethical approval was not required for this case report in accordance with institutional policy.

## Consent

Written informed consent for publication of clinical details and images was obtained from the patient.

## Conflicts of Interest

The authors declare no conflicts of interest.

## Supporting information


**Appendix S1:** Complete search strategy for all databases.
**Table S1:** Study‐level risk of bias assessment of included studies using JBI Critical Appraisal Checklists.

**Data S1:** PRISMA 2020 checklist.

## Data Availability

The data that support the findings of this study are available from the corresponding author upon reasonable request.
