# Supplementary material for: Atraumatic Subcapital Femoral Neck Fracture as the Initial Presentation of Osteonecrosis of the Femoral Head: A Case‐Based Systematic Review
Source: Orthop Surg. 2026 Oct 30:10.1111/os.70445. Online ahead of print. doi: 10.1111/os.70445 (PMC13720695; doi:10.1111/os.70445)
Supplement: Supplementary file 1 [file OS-9999-0-s001.docx]

**PRISMA 2020 Checklist**

**Atraumatic Subcapital Femoral Neck Fracture as the Initial Presentation of Osteonecrosis of the Femoral Head:
A Case-Based Systematic Review**

PROSPERO Registration: CRD420261342816 | Search date: February 28, 2026

Legend — Reported column: Yes = item fully addressed in the manuscript; Partial = item only partly addressed in the current manuscript; N/A = item not applicable or no formal assessment was performed because of the nature of the evidence.

| **Section** | **Item** | **Checklist Item** | **Located in Manuscript** | **Page** |
| --- | --- | --- | --- | --- |
| **TITLE** | 1 | Identify the report as a systematic review. | Title | 1 |
| **ABSTRACT** | 2 | See the PRISMA 2020 for Abstracts checklist. | Abstract (Objective, Methods, Results, Conclusions) | 1–2 |
| **INTRODUCTION** | 3 – Rationale | Describe the rationale for the review in the context of existing knowledge. | Introduction (§1, paragraphs 1–3) | 2–3 |
|  | 4 – Objectives | Provide an explicit statement of the objective(s) or question(s) the review addresses. | Introduction (§1, final paragraph) | 3 |
| **METHODS** | 5 – Eligibility criteria | Specify the inclusion and exclusion criteria for the review and how studies were grouped for the syntheses. | §3.1.2 Eligibility criteria | 8 |
|  | 6 – Information sources | Specify all databases, registers, websites, organisations, reference lists and other sources searched or consulted to identify studies. Specify the date when each source was last searched or consulted. | §3.1.3 Information sources and search strategy | 8 |
|  | 7 – Search strategy | Present the full search strategies for all databases, registers and websites, including any filters and limits used. | Appendix S1 (full search strings for PubMed, Embase, Cochrane CENTRAL, and Scopus) | 32 |
|  | 8 – Selection process | Specify the methods used to decide whether a study met the inclusion criteria of the review, including how many reviewers screened each record and each report retrieved, whether they worked independently, and if applicable, details of automation tools used in the process. | §3.1.4 Study selection | 9 |
|  | 9 – Data collection process | Specify the methods used to collect data from reports, including how many reviewers collected data from each report, whether they worked independently, any processes for obtaining or confirming data from study investigators, and if applicable, details of automation tools used in the process. | §3.1.5 Data extraction and risk of bias assessment | 9 |
|  | 10a – Data items | List and define all outcomes for which data were sought. Specify whether all results that were compatible with each outcome domain in each study were sought (e.g. for all measures, time points, analyses), and if not, the methods used to decide which results to collect. | §3.1.5; Tables 2–4 (extracted variables and treatment outcomes) | 9, 11–18 |
|  | 10b – Data items | List and define all other variables for which data were sought (e.g. participant and intervention characteristics, funding sources). Describe any assumptions made about any missing or unclear information. | §3.1.5; Tables 2–4; table footnotes document missing or non-disaggregated data | 9, 11–18 |
|  | 11 – Study risk of bias assessment | Specify the methods used to assess risk of bias in the included studies, including details of the tool(s) used, how many reviewers assessed each study and whether they worked independently, and if applicable, details of automation tools used in the process. | §3.1.5 (JBI checklists specified per study design); Table S1 | 9, 33–34 |
|  | 12 – Effect measures | Specify for each outcome the effect measure(s) (e.g. risk ratio, mean difference) used in the synthesis or presentation of results. | N/A – no comparative effect estimates; narrative synthesis only (§3.1.6) | N/A |
|  | 13a – Synthesis methods | Describe the processes used to decide which studies were eligible for each synthesis (e.g. tabulating the study intervention characteristics and comparing against the planned groups for each synthesis). | §3.1.6 Synthesis methods; §3.2.5 treatment-pooling criteria | 9, 14–15 |
|  | 13b – Synthesis methods | Describe any methods required to prepare the data for presentation or synthesis, such as handling of missing summary statistics, or data conversions. | §3.2.5 (exclusion of Guo et al. from treatment pooling); Table 4 footnote | 14–15, 18 |
|  | 13c – Synthesis methods | Describe any methods used to tabulate or visually display results of individual studies and syntheses. | Tables 2–4; Figure 5; Table S1 | 11–18, 30–31, 33–34 |
|  | 13d – Synthesis methods | Describe any methods used to synthesize results and provide a rationale for the choice(s). If meta-analysis was performed, describe the model(s), method(s) to identify the presence and extent of statistical heterogeneity, and software package(s) used. | §3.1.6 – narrative synthesis; meta-analysis not performed because comparative studies were absent | 9 |
|  | 13e – Synthesis methods | Describe any methods used to explore possible causes of heterogeneity among study results (e.g. subgroup analysis, meta-regression). | N/A – no meta-analysis or formal heterogeneity investigation; heterogeneity discussed narratively in §4.6 | N/A |
|  | 13f – Synthesis methods | Describe any sensitivity analyses conducted to assess robustness of the synthesized results. | N/A – narrative synthesis only; no sensitivity analyses were conducted | N/A |
|  | 14 – Reporting bias assessment | Describe any methods used to assess risk of bias due to missing results in a synthesis (arising from reporting biases). | No formal synthesis-level reporting-bias assessment was performed; potential publication/selective-reporting bias is discussed narratively in §3.2.6 and §4.6 | 15, 24–25 |
|  | 15 – Certainty assessment | Describe any methods used to assess certainty (or confidence) in the body of evidence for an outcome. | §3.1.5 (narrative certainty assessment and GRADE rationale); §3.2.7 Certainty of evidence | 9, 15 |
| **RESULTS** | 16a – Study selection | Describe the results of the search and selection process, from the number of records identified in the search to the number of studies included in the review, ideally using a flow diagram. | §3.2.1 Study selection; Figure 5 (PRISMA flow diagram) | 10, 30–31 |
|  | 16b – Study selection | Cite studies that might appear to meet the inclusion criteria but which were excluded, and explain why they were excluded. | A separate list of apparently eligible full-text reports excluded with reasons is not provided; aggregate exclusions are reported in §3.2.1/Figure 5 and eligibility criteria are specified in §3.1.2 | 8, 10, 30–31 |
|  | 17 – Study characteristics | Cite each included study and present its characteristics. | Table 2 (characteristics of all included studies) | 11–13 |
|  | 18 – Risk of bias in studies | Present assessments of risk of bias for each included study. | Table S1 (JBI appraisal for each included study) | 33–34 |
|  | 19 – Results of individual studies | For all outcomes, present, for each study: (a) summary statistics for each group (if appropriate) and (b) an effect estimate and its precision (e.g. confidence interval/credible interval), ideally using structured tables or plots. | Tables 2–4 (individual-study and synthesis data; comparative effect estimates were not available) | 11–18 |
|  | 20a – Results of syntheses | For each synthesis, briefly summarise the characteristics and risk of bias among contributing studies. | §3.2.3–3.2.6; Tables 3–4 | 14–18 |
|  | 20b – Results of syntheses | Present results of all statistical syntheses conducted. If meta-analysis was done, present for each the summary estimate and its precision and measures of statistical heterogeneity. If comparing groups, describe the direction of the effect. | N/A – narrative synthesis only (§3.1.6; §3.2.5) | N/A |
|  | 20c – Results of syntheses | Present results of all investigations of possible causes of heterogeneity among study results. | N/A – no formal heterogeneity investigation was performed | N/A |
|  | 20d – Results of syntheses | Present results of all sensitivity analyses conducted to assess the robustness of the synthesized results. | N/A – no sensitivity analyses were conducted | N/A |
|  | 21 – Reporting biases | Present assessments of risk of bias due to missing results (arising from reporting biases) for each synthesis assessed. | No formal synthesis-level reporting-bias assessment was performed; potential publication/selective-reporting bias is discussed narratively in §3.2.6 and §4.6 | 15, 24–25 |
|  | 22 – Certainty of evidence | Present assessments of certainty (or confidence) in the body of evidence for each outcome assessed. | §3.2.7 Certainty of evidence (overall certainty judged very low; formal GRADE not applied) | 15 |
| **DISCUSSION** | 23a – Discussion | Provide a general interpretation of the results in the context of other evidence. | §4 Discussion (main findings, diagnostic considerations, comparison with previous reports, treatment patterns, contralateral surveillance) | 19–25 |
|  | 23b – Discussion | Discuss any limitations of the evidence included in the review. | §4.6 Strengths, limitations, and future directions | 23–25 |
|  | 23c – Discussion | Discuss any limitations of the review processes used. | §4.6 Strengths, limitations, and future directions (heterogeneity, publication/selection bias, inconsistent staging and outcome reporting) | 23–25 |
|  | 24 – Conclusions | Provide a general interpretation of the results in the context of other evidence, and implications for future research. | §5 Conclusions | 25–26 |
| **OTHER INFO** | 25 – Registration and protocol | Provide registration information for the review, including register name and registration number, or state that the review was not registered. | §3.1.1 Protocol and registration (PROSPERO CRD420261342816) | 8 |
|  | 26 – Support | Describe sources of financial or non-financial support for the review, and the role of the funders or sponsors in the review. | Declarations – Funding: None | 27 |
|  | 27 – Competing interests | Declare any competing interests of review authors. | Declarations – Competing interests: None | 27 |
|  | 28 – Availability of data, code and other materials | Report which of the following are publicly available and where they can be found: template data collection forms; data extracted from included studies; data used for all analyses; analytic code; any other materials used in the review. | Declarations – Availability of data and materials | 27 |

Reference: Page MJ, McKenzie JE, Bossuyt PM, et al. The PRISMA 2020 statement: an updated guideline for reporting systematic reviews. BMJ. 2021;372:n71. doi:10.1136/bmj.n71
