# Supplementary material for: Atraumatic Subcapital Femoral Neck Fracture as the Initial Presentation of Osteonecrosis of the Femoral Head: A Case‐Based Systematic Review
Source: Orthop Surg. 2026 Oct 30:10.1111/os.70445. Online ahead of print. doi: 10.1111/os.70445 (PMC13720695; doi:10.1111/os.70445)
Supplement: Supplementary file 2 [file OS-9999-0-s002.docx]

**Appendix S1.** Complete search strategy for all databases.

| **Database** | **Search terms** |
| --- | --- |
| **PubMed** | ( ("Osteonecrosis"[Mesh] OR "Femur Head Necrosis"[Mesh]) OR osteonecrosis[tiab] OR "avascular necrosis"[tiab] OR "aseptic necrosis"[tiab] OR "bone necrosis"[tiab] OR "femoral head necrosis"[tiab] OR ONFH[tiab] OR AVN[tiab] ) AND ( "Femoral Neck Fractures"[Mesh] OR "femoral neck fracture"[tiab] OR "femoral neck fractures"[tiab] OR "subcapital fracture"[tiab] OR "subcapital fractures"[tiab] OR "cervical hip fracture"[tiab] OR "cervical hip fractures"[tiab] OR "intracapsular fracture"[tiab] OR "intracapsular fractures"[tiab] ) AND ( atraumatic[tiab] OR spontaneous[tiab] OR pathologic[tiab] OR pathological[tiab] OR insufficiency[tiab] OR nontraumatic[tiab] OR "non-traumatic"[tiab] OR "low-energy"[tiab] OR occult[tiab] ) |
| **Scopus** | osteonecrosis OR avascular necrosis OR aseptic necrosis OR bone necrosis OR femoral head necrosis AND femoral neck fracture OR subcapital fracture OR cervical hip fracture OR intracapsular fracture AND atraumatic OR spontaneous OR pathologic OR insufficiency OR nontraumatic |
| **Cochrane Central** | (osteonecrosis OR avascular necrosis OR aseptic necrosis OR bone necrosis OR femoral head necrosis):ti,ab,kw AND (femoral neck fracture OR subcapital fracture OR cervical hip fracture OR intracapsular fracture):ti,ab,kw AND (atraumatic OR spontaneous OR pathologic OR insufficiency OR nontraumatic):ti,ab,kw |
| **Embase** | (“osteonecrosis” OR “avascular necrosis” OR “bone necrosis” OR “femoral head necrosis" AND (“femoral neck fracture” OR “subcapital fracture” OR “intracapsular fracture”) AND (“atraumatic” OR “spontaneous” OR “pathological” OR “nontraumatic”) |

**Table S1.** Study-level risk of bias assessment of included studies using JBI Critical Appraisal Checklists**.**

| **Study** | **JBI tool used** | **Applicable items met** | **Overall appraisal** | **Main limitations / sources of bias** |
| --- | --- | --- | --- | --- |
| Usui et al. (1996) | Case series (10 items) | 6/10 (60%) | Moderate | Older series; incomplete recruitment method, timeline, and adverse-event reporting. |
| Kim & Kim (2000) | Case series (10 items) | 7/10 (70%) | Moderate | Small two-case report; limited consecutive inclusion and adverse-event detail. |
| Min et al. (2001) | Case series (10 items) | 8/10 (80%) | High | Retrospective series with limited long-term adverse-event reporting. |
| Loddenkemper et al. (2002) | Case report (8 items) | 5/8 (62.5%) | Moderate | Brief timeline and follow-up; limited adverse-event discussion. |
| Kamiya et al. (2004) | Case report (8 items) | 5/8 (62.5%) | Moderate | Limited chronological detail and outcome reporting. |
| Ko et al. (2004) | Case report (8 items) | 6/8 (75%) | High | Single-case design with limited adverse-event detail. |
| Lee & Suh (2005) | Case report (8 items) | 6/8 (75%) | High | Single-case design; limited patient-perspective and adverse-event reporting. |
| Zuckerman et al. (2006) | Case report (8 items) | 5/8 (62.5%) | Moderate | Limited timeline and adverse-event reporting. |
| Tompkins et al. (2010) | Case series (10 items) | 7/10 (70%) | Moderate | Small retrospective series; incomplete consecutive inclusion and follow-up detail. |
| Vaishya (2012) | Case report (8 items) | 5/8 (62.5%) | Moderate | Brief report with limited timeline and adverse-event reporting. |
| Fukui et al. (2015) | Case report (8 items) | 6/8 (75%) | High | Single-case design; limited external validity. |
| Kumar et al. (2016) | Case report (8 items) | 5/8 (62.5%) | Moderate | Limited chronological detail and adverse-event reporting. |
| Shah & Desai (2019) | Case report (8 items) | 6/8 (75%) | High | Single-case design; limited long-term follow-up. |
| Arora et al. (2021) | Case series (10 items) | 8/10 (80%) | High | Retrospective series without a comparator group. |
| Nishino et al. (2021) | Case report (8 items) | 6/8 (75%) | High | Single-case design; limited adverse-event reporting. |
| Xia et al. (2023) | Case report (8 items) | 6/8 (75%) | High | Single-case design; limited long-term follow-up. |
| Guo et al. (2023) | Cohort study (11 items) | 7/11 (63.6%) | Moderate | Retrospective subgroup analysis with confounding and non-disaggregated ONFH subgroup treatment. |
| Kwan et al. (2024) | Two-case report (10 items) | 8/10 (80%) | High | Two-case design; limited external validity. |

Abbreviations: JBI, Joanna Briggs Institute; ONFH, osteonecrosis of the femoral head. Overall appraisal thresholds were prespecified as >70% of applicable checklist items = high quality, 50–70% = moderate quality, and <50% = low quality. Items rated not applicable were excluded from the denominator.
